# Role of biochemical markers in the monitoring of COVID-19 patients

**DOI:** 10.5937/jomb0-29341

**Published:** 2021-03-12

**Authors:** Pablo Letelier, Nicole Encina, Pablo Morales, Alejandra Riffo, Halett Silva, Ismael Riquelme, Neftalí Guzmán

**Affiliations:** 1 Universidad Católica de Temuco, Facultad de Ciencias de la Salud, Departamento de Procesos Diagnósticos y Evaluación, Precision Health Research Laboratory, Temuco, Chile; 2 Universidad Autónoma de Chile, Facultad de Ciencias de la Salud, Instituto de Ciencias Biomédicas, Chile

**Keywords:** coronavirus disease, COVID-19, SARS-CoV2, laboratory diagnosis, biomarkers, korona virus oboljenje, COVID-19, SARSCoV-2, laboratorijska dijagnostika, biomarkeri

## Abstract

COVID-19 is an infectious disease caused by the SARSCoV-2 virus, which has given rise to a global sanitary emergency. The clinical characteristics of COVID-19 are varied and can range from an asymptomatic infection to a mild to severe pneumonia. Recent studies have shown that different laboratory parameters become altered in these patients, and as such are useful as biomarkers to assess the progression of the disease and categorize patients that may present a severe and/or fatal clinical condition. This review analyzes biochemical and immunological markers that become altered in COVID-19 patients and their impact on different organs at a hepatic, cardiac, renal and pancreatic level, as well as markers of inflammation, analyzing their implications in the evolution of the disease.

## Abstract (sr)

COVID-19 je infektivno oboljenje izazvano SARS CoV-2 virusom, koje je dovelo do globalne pandemije. Kliničke karakteristike COVID-19 su različite i kreću se od asimptotske infekcije do srednje ili teške pneumonije. Nedavne studije ukazuju da se različiti laboratorijski parametri menjanju kod ovih pacijenata, i kao takvi su korisni kao bio-markeri za praćenje napredovanja oboljenja i kategorizaciju pacijenata koji mogu imati teška i/ili fatalna klinička stanja. U ovom radu su analizirani biohemijski i imunološki markeri koji se menjaju kod COVID-19 pacijenata i pokazatelj su stanja različitih organa kao što su jetra, srce, bubrezi, pankreas, i koriste se kao markeri inflamacije, a za potrebe procene napredovanja i toka oboljenja.

## Introduction

In December 2019, the city of Wuhan in China became the epicenter of unexplainable cases of pneumonia, which in January 2020 were identified as a new coronavirus, turning rapidly into a major problem of public health worldwide [1]. This pathogen, corresponding to a beta coronavirus, is made up of single chains of positive RNA belonging to the large Coronaviridae subfamily and has the ability to infect mammals and other animals [2]. The 2019 coronavirus disease (COVID-19) is caused by the coronavirus 2 of the severe acute respiratory syndrome (SARS-CoV-2) [3], which spread rapidly all over the world and was declared by the World Health Organization (WHO) as a pandemic on 11 March 2020 [4].

The entry path of SARS coronavirus into the host cells has been associated fundamentally with the angiotensin II-converting enzyme (ACE2) that, when is located in the membrane of the cell, can function as a receptor for certain proteins expressed by this virus. The tissue location of ACE2 would be related to how the disease affects different tissues or organs [5]. This protein is expressed predominantly in different cell types such as epithelial, pulmonary alveolar, small intestine epithelial, vascular endothelial and smooth muscle cells [5]. ACE2 regulates the angiotensinrenin system, counteracting the activity of the angiotensin-converting enzyme (ACE) and having a protective effect on acute respiratory distress syndrome (ARDS). ACE2 is a type I transmembrane glycoprotein consisting of 805 amino acids and a single extracellular catalytic domain with a molecular weight of approximately 120 kDa, whereas the ACE2 coding gene is located in chromosome X [6]. Similar to ACE, ACE2 has two domains: the amino-terminal catalytic domain and the carboxy-terminal domain. The catalytic domain exhibits 41.8% sequence identity with the amino domain of ACE [6].

The coronavirus family contains seven viruses that can cause severe infections in human beings: HCoV-229E, HCoV-HKU1, HCoV-NL63, HCoV-OC43, SARS-CoV, MERS-CoV and SARS-CoV-2. These are zoonotic viruses with single-stranded enveloped RNA, spherical in shape with pronounced protrusions of spike glycoproteins, called protein S, on the surface of the envelope, in addition to structural proteins including the envelope (protein E), the matrix (protein M), and the nucleocapsid (protein N) [7] [8]. The spikes (S) glycoproteins of the virus act as binding proteins and are thus highly important because they allow the entry of this virus into the target cell. Protein S is trimeric, equipped with subunits S1 and S2, where S1 carries the receptor-binding domain (RBD) that interacts with the target cell and binds with an ACE2 domain [9]. When interacting it produces a greater concentration of angiotensin II and can unleash local vasoconstriction. It also binds with pulmonary and blood vessel cell receptors, which in severe cases leads to pulmonary compromise and diffuse alveolar damage, coating them with a membrane and causing respiratory distress and fibrosis [10].

COVID-19 is diagnosed through laboratory tests in patients with epidemiological history and clinical symptoms, together with radiological examinations [11] [12]. The COVID-19 disease has different clinical presentations, ranging from asymptomatic to mild, moderate or severe symptoms, with or without the presence of pneumonia [13]. Fever and coughing are the most common symptoms at a global level [14].

The accumulated evidence has shown that many biochemical parameters become altered in COVID-19 patients, and this has been correlated with the severity of the disease and in some cases associated with the prognosis of the patients. The laboratory parameters together with other demographic and clinical data of patients could allow them to be categorized in the initial stages, thus identifying people who will become critically ill and making it possible to improve their clinical care and seek adequate therapeutic strategies. This paper focuses on analyzing the importance of biochemical biomarkers (hepatic, cardiac, renal, pancreatic and inflammatory) in COVID-19 patients and their implications in the evolution of the disease.

## Role of the laboratory in the assessment of biochemical parameters in COVID-19 patients

Clinical laboratories play an essential role in the detection of the virus as well as to the follow-up of patients (monitoring their evolution) and epidemiological surveillance via the determination of serological markers in their systems [15]. Laboratory tests validated for SARS-CoV-2 are crucial for the timely management of COVID-19 because they support the clinical decision-making process for controlling infections and detecting asymptomatic cases. This expedites speedy isolation, adequate treatment and consequently reduces contagion rates [16].

Many laboratory parameters make it possible to assess the severity of the disease and predict the risk of it evolving toward more serious afflictions such as acute respiratory distress syndrome (ARDS), disseminated intravascular coagulation (DIC) and multiple organ failure (MOF) [17]. Some parameters for which an unfavorable course of the disease has been described are absolute neutrophilia, thrombocytopenia, hypoalbuminemia, the elevation of liver enzymes, creatinine and nonspecific inflammatory markers such as C-reactive protein (CRP) and Interleukin 6 (IL-6) [18]. Not with standing the above, the main progression predictors described are lymphopenia, elevated D-dimer and hyperferritinemia, although it is also necessary to consider LDH, CPK and troponin in the marker panel [19].

## Inflammatory response markers

Table 1 presents the laboratory findings of parameters associated with inflammation when admitting patients diagnosed with COVID-19, depending on the severity of the clinical condition. An exacerbated reaction of the immune system in COVID-19 patients provokes an inflammatory response called »cytokine storm«, which cause damage to different tissues contributing to worsening the condition of the patient [20] [21]. Lymphopenia and elevated proinflammatory cytokines have been reported to be frequent in severe cases of COVID-19 in comparison with milder cases, with higher serum concentrations of IL6, IL10, IL2 and IFN-γ present in severe cases [22]. In this context, it has been observed that the neutrophil-to-lymphocyte ratio (NLR) and neutrophil-to-CD8+ T cell ratio (N8R) can be useful as diagnostic factors in seriously ill patients [22]. In addition, it was established that cytokines (IL-2, IL-4, IL-10, IFN-γ and TNF-α), except for IL-6, show maximum levels in serum 3 to 6 days after the onset of the disease. Both the IL-6 and IL-10 levels displayed significant increases in the seriously sick group in comparison with the milder cases and significant increases in the serum levels of IL-2 and IFN-γ were only observed in the seriously ill group 4 to 6 days after the onset of the disease [22].

**Table 1:** Laboratory parameters related to inflammation on admission of patients diagnosed with COVID-19, according to the severity of the disease Each value of the table represents the mean, median; –: Data not available; M: Male; F: Female; S: Statistically significant; NS: Not statistically significant(+): proteinuria or hematuria +, ++ or +++. *Other classifications*: R: Recovered; D: Deceased; NLF: Normal liver function; ALF: Abnormal liver function

Parameter	N° of cases	Reference range	Severity of the Disease				* P value *	Reference
			Total cases	Mild cases	Serious cases	Other classifications		
Lactate dehydrogenase (U/L)	138	125–243	261	212	435		S	[23]
	99	120–250	336	–	–		–	[24]
	41	≤245	286	281	400		S	[25]
	40	–	304	221	462		NS	[22]
	12	114–240	605	–	–		–	[26]
	19	120–250	256	–	–		–	[27]
	174	M: 135–225<br> F: 135–214	267	246	336		–	[28]
	701	–	377	–	–	NLF: 364; ALF: 458	S	[29]
	150	120–250	–	–	–	R: 289; D: 906	S	[30]
	148	–	224	–	–	NLF: 201; ALF: 257	S	[31]
C-reactive protein (mg/L)	73	0–5.0	51.4	–	–		–	[24]
	12	<10	41.1	–	–		–	[26]
	19	0–4	26.5	–	–		–	[27]
	136	0–5	34.2	28.7	46.6		S	[32]
	452	0–1.0	44.1	33.2	57.9		S	[33]
	40	–	38.1	7.6	62		–	[22]
	157	<1	31.9	33.95	19.31		–	[28]
	150	0–5.0	–	–	–	R: 34; D: 126	S	[30]
	148	–	17.7	–	–	NLF: 13; ALF: 25	S	[31]
Procalcitonin (ng/mL)	138	<0.05	49	22	27		S	[23]
	99	0–5.0	0.5	–	–		–	[24]
	41	<0.1	0.1	0.1	0.1		–	[25]
	12	0–0.5	0.81	–	–		–	[26]
	118	0–5	0.07	0.05	0.1		S	[32]
	452	0–0.05	0.1	0.05	0.1		S	[33]
	148	–	0.3			NLF: 0.02; ALF: 0.06	S	[31]
Interleukin-1β (pg/mL)	452	0–5.0	5.0	5.0	5.0		NS	[33]
Interleukin-6 (pg/mL)	99	0–7	7.9	–	–		–	[24]
	7	0–7	19.3	–	–		S	[27]
	452	0–7	21.0	13.3	25.2		S	[33]
	150	0–7	–	–	–	R: 6.8; D: 11.4		[30]
Interleukin-10 (pg/mL)	452	0–9.1	5.4	5.0	6.6		S	[33]

Huang et al. [25] established that the plasma concentrations of IL2, IL7, IL10, GCSF, IP10, MCP1, MIP1A, and TNF-α were higher in Intensive Care Unit (ICU) patients in comparison with non-ICU patients. In turn, Qin et al. [33] reported that a majority of severe cases showed an elevation of biomarkers related to infection (procalcitonin, serum ferritin and CRP) and inflammatory cytokines (IL-2R, IL-6, IL-8, IL-10 and TNF-α).

C-reactive protein is a plasma protein that is synthesized by the liver and induced by different inflammatory mediators such as IL-6. Despite being nonspecific, it is used clinically as a biomarker for different inflammatory complaints, and an increase in its levels is associated with greater severity of the disease [34]. This protein can activate the complement through the classic route and has the ability to modulate the function of phagocytic cells, properties that suggest it would play a role in the opsonization of infectious agents and damaged cells [35]. In a study where the levels of C-reactive protein in COVID-19 patients were evaluated and categorized in four stages according to the computed tomography (CT) findings: initial (3 days), progression (7 days), peak (12 days) and recovery (16 days), the results of the seriously ill group (n=6) presented higher levels of CRP in the progression stage than the milder group (n=21), but decreased without statistically significant differences in the peak and recovery stages [36]. Moreover, Liu et al. [37] established that the C-reactive protein was significantly higher in the progression group than in the recovery/stabilization group (38.9 vs. 10.6 mg/L). In contrast, albumin diminished significantly in the progression group (41.27 g/L) in comparison with the recovery group (36.62 g/L) [37]. In the multivariate analysis both the albumin (Odds ratio, OR) 7.353, confidence interval (CI) 95%: 1.098-50.000; P level= 0.003) and CRP (OR, 10.530; CI95%: 1.224-34.701, P= 0.028) were risk factors for the progression of the disease. The plasma concentrations of these proteins in the acute phase (albumin, CRP) become modified by at least 25% in response to certain cytokines produced during different types of inflammatory processes where some degree of tissue damage is involved [38] [39].

Furthermore, it has been reported that the elevation of the lactate dehydrogenase enzyme (LDH) used as a marker of lung tissue damage, is one of the most frequent biochemical anomalies in COVID-19 patients on admission to the hospital [15]. Many studies have shown that patients with mild infection have LDH values within the reference ranges, unlike patients in critical condition [22] [31] [23], where a significant difference in the magnitude of the alteration is observed in the seriously ill patients [25] [29] [28] [30].

Yuan et al. [40] revealed that in the first six days of hospitalization, those patients in severe clinical conditions showed significantly higher levels of IL-6 and LDH in serum than those of the moderate group, which descended drastically around day 6 to 9 in both groups of patients. Interestingly, the decrease of serum levels of LDH and CK was correlated with the elimination of viral mRNA, suggesting that the constitutive reduction in the levels of LDH or CK is likely to predict a favorable response to the course of infection in COVID-19 patients [40].

In patients with severe pneumonia, the initial infectious condition precedes (between 5 and 9 days) a state of systemic inflammatory hyper reactivity, probably favored by the »cytokine storm« or macrophage activation syndrome (MAS). This typically occurs in subjects with ARDS, where the exacerbated CRP increase and hyper ferrfitinemia are key for diagnosing MAS and are elevated in many serious cases of pneumonia due to COVID-19 [41].The low survival rate of ARDS has been related to a sustained elevation of IL-6 and IL-1 [41]. This hyper inflammatory state is characterized by increased acute phase reactants associated with an exacerbated increment of pro-inflammatory cytokines such as IL-6, clearly elevated in non-survivors in comparison with those who survive throughout the clinical course and increasing with the deterioration of the disease [42]. In the univariate analysis, it was possible to associate it with a greater probability of in-hospital death in patients with elevated LDH, IL-6 and procalcitonin [42], where it was observed that LDH increases in both groups (survivors and non-survivors) in the early stage of the disease, but in this study decreased as of day 13 in the survivors [42].

Extra pulmonary systemic hyper inflammation syndrome occurs in a minority of infected patients and is characterized by the presence of so-called »cytokine storm«, induced by increased levels of cytokines and interleukins such as IL-2, IL-6, IL-7, IL-10 and tumor necrosis factor (TNF) α, together with several other biomarkers such as granulocyte colonystimulating factor (G-CSF), interferon-inducible protein 10 (IFN), monocyte chemoattractant protein 1 (MCP-1) and inflammatory macrophage protein 1-α. On the other hand, this pathology is also characterized by a decrease of CD4+ and CD8+ T cells, and of the IFN-γ expression by CD4+ T cells [43]. Moreover, higher levels of G-CSF have been also found in ICU patients, which is associated significantly with severe disease [44].

## Cardiac markers

The risk factors associated with cardiac events during the COVID-19 disease include advanced age, preexisting cardiovascular disease and greater severity in the presentation of pneumonia. The elements through which these events occur include: 1) systemic mechanisms such as pro-inflammatory responses of the atherosclerosis-mediating cytokines (IL-6, IL-7, IL-22, CXCL10), which contribute directly to plaque rupture through local inflammation; 2) induction of pro-coagulation factors; and, 3) hemodynamic changes predisposing to ischemia and thrombosis [42]. For this reason, the comorbidities and direct and indirect cardiac damage contribute to producing slight increases in the high-sensitivity troponin I markers (hsTn) or the N-terminal pro B type natriuretic peptide (NT-proBNP) in hospitalized patients [45].

Table 2 presents the laboratory findings of parameters associated with cardiac, hepatic and renal function on the admission of patients diagnosed with COVID-19, according to the severity of the clinical condition. ACE2 expression has been detected in cardiac endothelial cells, vascular smooth muscle cells and cardiomyocytes [46], suggesting that SARS-CoV-2 could enter the host cells via the binding of the spike protein with ACE2, affecting cardiovascular homeostasis through the renin-angiotensin-aldosterone system (RAAS) and generating a myocardial injury [47]. Previously, cardiac tissue biopsy had revealed the presence of SARS-CoV-1 in 35% of deceased subjects [48].

**Table 2:** Laboratory parameters related to cardiac, hepatic and renal function on the admission of patients diagnosed with COVID-19, according to the severity of the disease Each value of the table represents the mean, median –: not available; M: Male, F: Female; S: statistically significant difference between mild and severe cases N; S: non–significant difference between mild and severe cases (+): proteinuria or hematuria +, ++ o +++; **: upper BUN limit according to age <60, 60–80 and >80 years; for men it is 8.0, 9.5 and 8.3 mmol/L respectively, and for women 7.5, 8.8 and 8.3 mmol/L respectively. *Other classifications*: R: Recovered; D: Dead; NScr: Normal base serum creatinine; EScr: Elevated base serum creatinine; NLF: Normal liver function; ALF: Abnormal liver function; LI: Liver injury

Parameter	N° of ses	Reference range	Severity of the disease				* P value *	Reference
			Total cases	Mild cases	Serious cases	Other classifications		
Troponin I (pg/mL)	138	<26.2	6.4	5.1	11		S	[23]
	150	2–28	–			R: 3.5; D: 30.3	S	[30]
	41	≤28	3.4	3.5	3.3		–	[25]
Troponin I (µg/mL)	12	0–0.1	0.95	–	–		–	[26]
Creatine kinase-MB (U/L)	138	<25	14	13	18		S	[23]
Creatine kinase-MB (ng/mL)	12	0–2.37	2.0	–	–		–	[26]
Myoglobin (ng/mL)	99	0–146.9	67.9	–	–		–	[24]
	12	0–110	43.9	–	–		–	[26]
	65	0–106	58.6	21.6	63.4		–	[28]
	150	0–146.9	–			R: 77.7; D: 258.9	S	[30]
Natriuretic peptide (pmol/L)	4	0–23.1	43.9	–	–		–	[26]
Alanin aminotransferase (U/L)	138	9–50	24	23	35		S	[23]
	99	9–50	39	–	–		–	[24]
	41	–	32	27	49		S	[25]
	40	–	22.5	19	27		–	[22]
	12	0–45	31.6	–	–		–	[26]
	105	M: 9–50 F: 7– 40	23.5	22	27.8		S	[49]
	18	9–50	36.4	–	–		–	[27]
	193	M: 0– 41 F: 0–33	20	19	21		–	[28]
	150	9–50	–			R: 48.7; D: 170.8	–	[30]
	701	–	35			NScr: 36; EScr: 32	–	[29]
	417	≤40	21			NLF: 17; ALF: 27; LI: 47	S	[50]
Aspartataminotransferase (U/L)	138	15–40	31	29	52		S	[23]
	99	15–40	34	–	–		–	[24]
	41	≤40	34	34	44		NS	[25]
	12	0–45	40	–	–		–	[26]
	50	M: 15–40 F: 13–35	24.2	22	46.3		S	[49]
	18	15–40	34.9	–	–		–	[27]
	193	M: 0–40 F: 0–32	30	26	40		–	[28]
	40	–	34.1	25.9	51.2		–	[22]
	150	15–40	–			R: 48.7; D: 288.9	NS	[30]
	701	–	42			NScr: 41; EScr: 47	NS	[29]
	417	≤40	26.5			NLF: 23; ALF: 34; LI: 47	S	[50]
Gamma-glutamyltranspeptidase(U/L)	14	7–45	42.2	–	–		–	[27]
	417	≤49	34.1			NLF: 21; ALF: 36; LI: 135	S	[50]
Total bilirubin (mmol/L)	138	5–21	9.8	9.3	11.5		S	[23]
	701	–	12			NScr: 11; EScr: 21	NS	[29]
	41	–	11.7	10.7	14		S	[25]
Total bilirubin (µmol/L)	50	0–18.8	10.2	10	10.6		NS	[49]
	12	3–22	8.2	–	–		–	[26]
	40	–	10.3	8.8	13.2		–	[22]
	193	M: 0–26 F: 0–21	8.82	7.85	13.39		–	[28]
	99	0–21	15	–	–		–	[24]
	150	0–26	–			R: 5.1; D: 8.6	S	[30]
	417	≤17.1	10.9			NLF: 9; ALF: 17; LI: 17	S	[50]
Alkaline phosphatase (U/L)	417	≤135	61			NLF: 59; ALF: 63; IL: 68	S	[50]
Albumin (g/L)	99	40–55	31.6	–	–		–	[24]
	41	–	31.4	34.7	27.9		S	[25]
	12	40–55	37.7	–	–		–	[26]
	49	40–55	41.6	42	37.2		S	[49]
	193	35–52	34.2	35.2	32.6		–	[28]
	150	35–52	–			R: 33; D: 28.8	S	[30]
Blood ureanitrogen (mmol/L)	138	2.8–7.6	4.4	4.0	5.9		S	[23]
	99	3.6–9.5	5.9	–	–		–	[24]
	12	3.2–7.1	5.4	–	–		–	[26]
	87	2.9–8.2	3.8	3.72	4.14		NS	[51]
	193	3.1–8	4.4	3.9	6.43		–	[28]
	40	–	3.2	3.2	3.3		–	[22]
	701	**	5.7			NScr: 4.8; EScr: 11	S	[29]
	150	3.1–8	–			R: 5.1; D: 8.65	S	[30]
Serum creatinine (µmol/L)	138	64–104	72	71	80		S	[23]
	99	57–111	75.6	–	–		–	[24]
	41	≤133	74	73	79		NS	[25]
	12	58–110	85.6	–	–		–	[26]
	40	–	67.3	64.0	74.2		–	[22]
	87	M: 54–133 F: 44–106	65.2	65.3	71		NS	[51]
	193	M: 59–104 F:45–84	66	63	73		–	[28]
	701	M: 104 F: 84	77			NScr: 68; EScr: 132	S	[29]
	150	59–104	–			R: 72.1; D: 91.2	S	[30]
Proteinuria (+)	83	Negative	35%	28%	58%			[51]
	129	Negative	59%	55%	66%			[28]
	442	Negative	44%			NScr: 40%; EScr: 70%		[29]
Hematuria (+)	83	Negative	29%	22%	53%			[51]
	129	Negative	44%	44%	45%			[28]
	442	Negative	27%			NScr: 23%; EScr: 53%		[29]

A meta-analysis of 4,189 patients in 28 studies established that seriously ill COVID-19 patients presented significantly higher levels of troponin, creatine kinase-MB (CK-MB), myoglobin and NT-proBNP. Acute cardiac injury (troponin elevation), was more frequent in patients with serious conditions in comparison with their milder counterparts. Predominantly, hsTnI and NT-proBNP levels increased during the course of the hospitalization solely in non-survivors [52].

Churchill et al. [53] evaluated the prevalence and reversibility of left ventricular dysfunction using echocardiography, including 93 patients with a comorbidity history of hypertension (60%), diabetes mellitus (41%) and obesity (50%). 88% of the patients had to be admitted to the ICU with the support of mechanical ventilation and 71% required vasopressor support. In these patients (n=93) the median of highsensitivity troponin (hsTn) was 51 ng/L and that of the natriuretic peptide was 1.643 pg/mL. Of the subset of 50 patients with a troponin level of 50 ng/L, 48% evidenced left ventricular dysfunction [53].

In turn, Huang et al. [25] found that hsTnl increased substantially in five patients (12.2%), who were diagnosed with cardiac injury related to the virus. It was also observed that the aspartate aminotransferase (AST) enzyme increased in 62% of the ICU patients compared to an increase in only 25% of those patients not in the ICU [25]. In another 138 patients, the average levels of hsTnl were within the reference range, although they increased in ICU patients [23]. It has also been reported that the maximum level of cardiac troponin I (0.00 ng/L vs. 0.56 ng/L; p<0.01) and the maximum levels of NT-proBNP (301.2 ng/L vs 2887.5 ng/L; p<0.01) and creatine kinase MB (1.3 ng/mL vs 3.9 ng/mL; p<0.01) were higher in patients admitted to the ICU or who required mechanical ventilation, extracorporeal membrane oxygenation (ECMO) or who died. In addition, the Cox proportional hazard ratio analysis showed that the maximum level of cardiac troponin I and the maximum level of NT-proBNP have presented significantly higher risk ratios (8.9 (CI 95%: 1.9-40.6; p<0.01) and 1.2 (95% CI: 1.1-1.3; p<0.01)), respectively [54]. Moreover, in the univariate analysis Zhou et al. [42] associated greater likelihood of death in patients with elevated ALT, high-sensitivity cardiac troponin I and creatine kinase, while in the group of non-survivors the high-sensitivity cardiac troponin I increased rapidly as of day 16 after the onset of the disease. In the same context, Deng et al. [54] established that a majority of patients had normal levels of troponin on admission but, in 37.5% of the cases, these levels increased during hospitalization particularly in those patients who then died. In fact, the troponin levels increased significantly within that week before death [54].

In the study by Chen et al. [24] it was observed that 76% of patients (n=99) had an abnormal myocardial zymogram, with CK elevation in 13 patients and LDH elevation in 75 patients, one of whom also exhibited abnormal CK (6280 U/L) and LDH (740 U/L).

### Hepatic markers

Elevation of alanine aminotransferase (ALT), AST and total bilirubin is common in seriously ill patients, who present more frequent signs of liver dysfunction than those with milder conditions [44]. In the Chen et al. [24] study, where 99 confirmed cases of COVID-19 were included, it was observed that 43 patients presented liver dysfunction, where ALT or AST rose above the reference range and, in particular, one case presented serious hepatic function damage with ALT and AST values of 7590 U/L and 1445 U/L, respectively.

Cai et al. [50] established that over 90% of patients with abnormal hepatic tests were mild on admission (i.e., with<2 × upper limit of normal (ULN), and around 24% of them exhibited ALT and GGT elevations of more than three times the upper limit during hospitalization. However, the increase in AST and total serum bilirubin (TBIL) to more than 3 x ULN was moderate (12% and 15%, respectively), and no increase in ALP was found. Patients with abnormal hepatocellular or mixed type hepatic tests on admission were more likely to evolve toward a serious illness (OR 2.73; CI 95%: 1.19 to 6.3 and 4.44; CI 95%: 1.93 to 10.23, respectively). Liver damage during hospitalization was especially associated with the use of medication such as lopinavir and ritonavir [50].

Gong et al. [34] evaluated 12 patients displaying alterations in laboratory parameters such as hypoalbuminemia, lymphopenia and neutrophilia, increased CRP and LDH, and reduced CD8+ count, concluding that the combination of hypoalbuminemia, lymphopenia and high concentrations of CRP and LDH could predict a more serious acute lung injury on admission to the hospital.

A post-mortem hepatic histopathological analysis in a 51-year-old man who died as a result of severe acute respiratory syndrome due to coronavirus, showed moderate microvesicular steatosis and mild lobular and portal activity, indicating that this liver injury could have been caused by SARS-CoV-2 infection or induced by medication [55]. In addition, the inflammation mediated by the immune system, such as the cytokine storm and hypoxia associated with pneumonia could also have contributed to the liver injury, eventually producing hepatic insufficiency in seriously ill patients [56]. This occurs because the hypoxia and shock induced by the complications (such as respiratory distress syndrome, systemic inflammatory response syndrome and multiple organ failure) can also cause hepatic ischemia and hypoxiareperfusion dysfunction [57]. This finding correlates with previous reports of autopsies performed on patients with SARS-CoV-2 [58].

## Renal markers

Reports have stated that, in the kidneys, ACE-2 is highly expressed on the brush border of the proximal tubular cells and to a lesser extent in the podocytes. In contrast, its expression in glomerular endothelial and mesangial cells has not been described [46]. Renal disease in COVID-19 patients can manifest itself in the form of acute renal injury (ARL), hematuria or proteinuria, promoting a greater mortality risk. It is unclear whether ARL is due largely to hemodynamic changes and cytokine release or the direct toxicity of the virus [59].

Renal histopathological examinations on six patients who died of COVID-19 in Wuhan, China, showed deterioration of the renal function, in addition to different degrees of acute tubular necrosis, detachment of the luminal brush border and degeneration of the vacuole in different areas of the six samples. As a whole, these results established that the infection mainly induces severe acute tubular necrosis and lymphocyte infiltration. The viral antigen of the nucleocapsid protein (NP) was studied by immunohistochemistry, revealing its presence in the tubules of all the renal tissue samples [60]. Although acute renal failure has been reported particularly in seriously ill patients, it has also been observed that both severe and milder cases display normal levels of serum creatinine and cystatin C (n=178). Even though 23.6% of the patients showed a reduced estimated glomerular filtration rate (eGFR), only 2.8% of them exhibited a high BUN level and none presented an increase of serum creatinine. According to these results, none of the patients developed an acute renal injury or acute renal insufficiency during their entire hospitalization, regardless of their admission to the ICU [51]. Interestingly, of the 83 patients without any kidney disease history who were subjected to a routine urine test when hospitalized, 45 (54.2%) exhibited an abnormal result, such as proteinuria, hematuria and leukocyturia, but none presented acute renal insufficiency (ARI) throughout the study. Probably, these renal alterations may have been due to the nephro-toxicity of medication used before hospital admission, although the possibility that they were produced by the viral infection cannot be discarded [51]. In line with other reports, a low degree of renal function damage, 7.32% (7 of 99), was also observed [24].

In contrast, an analysis of 59 cases hospitalized for COVID-19 in Wuhan (including 28 diagnosed as severe cases and 3 who died), found that 63% (32 of 51 patients) presented proteinuria, of which a large number (64%) was found to have the detected urine protein on the first day of admission, suggesting that renal impairment was already present before or at the time of admission. 27% (16 of 59 patients) of the patients had a high level of ureic nitrogen and the blood urea nitrogen (BUN) levels in 23 cases showed that 43% of these experienced an increase within an interval of 2 to 10 days, and 2/3 of the non-surviving patients exhibited notably high levels before their death. 19% (11 of 59 patients) also exhibited a high level of creatinine. Lastly, the computed tomography results of all patients (100%) revealed a radiographic abnormality in the kidneys [28].

Cheng et al., when evaluating a group of 701 hospitalized patients, showed that 44% had proteinuria and hematuria, while at least 26.7% presented hematuria on admission, with a prevalence of high creatininemia and uremia of 15.5% and 14.1%, respectively. During the study period, 3.2% of the patients developed ARI [29]. In addition, the Cox regression showed that the elevated levels of base serum creatinine (>133 µmol/L) and BUN, plus acute kidney injury (AKI) higher than stage 2, proteinuria of any degree and hematuria of any degree are independent risk factors of in-hospital death [29]. Moreover, Wang et al. [23] reported that as the disease advanced and the clinical condition deteriorated, the levels of urea and creatinine in the blood increased progressively before death, reaching significant levels (p<0.05) as of day 13 and 17 respectively after the onset of the disease. The results support the idea that kidney damage is common in COVID-19 patients and can be one of the main causes of the severity level of the disease, contributing to multiple organ failure and death [61]. Therefore, monitoring of the kidney function would be fundamental in the clinical management of infected patients, as well as early treatment of renal failure with continuous therapy.

## Pancreatic markers

Mild pancreatic injury has been reported in some COVID-19 patients [62], which can be explained at least partly by different mechanisms that include the direct cytopathic effects of SARS-CoV-2, as well as indirect and immune-mediated systemic inflammatory cell responses. Moreover, the SARS-CoV-2 receptor of angiotensin-converting enzyme 2 is highly expressed in cells of the pancreatic islets and exocrine glands, which can cause the infection to damage the islets and result in acute diabetes [62] [63] [64], thus rendering the quantification of pancreatic enzymes such as amylase or lipase useful for followup purposes [62].

The post-infection pancreatic injury was analyzed in a study that included 212 patients. In mild cases, 1.85% (1 of 54) presented increased levels of amylase and lipase, where as he severe cases presented 17.91% (12 of 64) and 16.41% (11 of 64) of amylase and lipase, respectively. In computed tomography, 5 seriously ill patients (7.46%) exhibited changes, mainly focal enlargement of the pancreas or dilation of the pancreatic duct, without acute necrosis [65]. In this context, a study described the presence of acute pancreatitis associated with SARS-CoV-2 in members of a family, ruling out other causes of pancreatitis. In one of the cases (a 47-year-old woman) pancreatic amylase was 173 U/L on admission, rising rapidly to > 1500 U/L after 11 hours. In a second case (68-year-old woman), the amylase level increased from 85 U/L on admission day to 934 U/L on day six, and the modified Glasgow acute pancreatitis severity score was 5 points, consistent with severe acute pancreatitis [66].

Wang et al. [62] reported that 17% of patients (n=52) with pneumonia due to COVID-19 showed high values of amylase or lipase, with five patients presenting underlying disorders such as hypertension, diabetes and heart disease. It is known that an elevation in pancreatic enzymes can be produced by other gastrointestinal disorders, and these have also been reported in COVID-19 patients [67], which led De-Madaria et al [68]. to suggest that pancreatic injury in COVID-19 patients be established based on imaging criteria such as computed tomography or magnetic resonance.

## Discussion

COVID-19 constitutes a major public health problem at a global level and is related to multiple complications that affect both the health of patients and the associated health care costs. For this reason, clinical laboratories can contribute to establishing biomarkers that would make it possible to stratify the risk of patients evolving toward more serious conditions, thus expediting clinical decision making [23] [69]. Although predisposing factors have been defined (gender, risk ages and association with pathologies such as obesity, diabetes and hypertension) [42], it is fundamental to use biomarkers that would allow a distinction to be made regarding the progression of the disease and anticipate which patients could require advanced medical procedures, thus making possible a focalized use of clinical resources.

The available evidence shows that patients with severe and critical cases present various alterations in their laboratory parameters [23], and in some cases rapidly evolve toward ARDS and septic shock followed by multiple organ failure [24]. In this context, high levels of IL-6 and increased concentrations of highsensitivity troponin I have been described as frequent in seriously ill COVID-19 patients [70].

Kidney function biomarkers such as serum creatinine were in general observed to be within the reference range [22] [25] [23] [28] [30] [24] [26] [51] and no statistically significant differences were found between the mean values of patients with mild and severe conditions on admission (Table 2). During the time of hospitalization, however, it was observed that the non-surviving patients had a progressive increase (approximately as of day 10 after admission) above the reference range, reaching a peak a few days before they died. BUN levels behaved similarly, increasing during the hospitalization period in seriously ill patients. Therefore, these parameters could be used to assess the prognosis of patients in the course of their hospitalization. Renal dysfunction in nonsevere COVID-19 cases was minor and was not diagnosed as ARI, unlike patients with severe cases, where 43 out of 65 were diagnosed with ARI [28]. Patients with end-stage renal disease (ESRD) are particularly vulnerable to severe COVID-19 due to the advanced age and high frequency of comorbidity such as diabetes and hypertension in this population [59].

Although respiratory symptoms are predominant in COVID-19, the cardiac pathology is of special interest because it increases both the risk of infection due to SARS-CoV-2 and the severity of the condition. Cardiac insufficiency is one of the most frequent and severe complications of SARS-CoV-2 because increases the death risk by causing rapidly progressive fulminant myocarditis [30]. The cardiac problems can continue even after recovery, regardless of any preexisting condition, severity level and general course of the acute illness or time elapsed since the original diagnosis [71]. The damage caused in severe cases of the disease is reflected in the elevation of biomarkers such as troponin I, CK-MB, BNP and myoglobin. Although during admission the values of these parameters are observed to be normal in mild and serious cases in patients, an exacerbated increase of these cardiac markers is observed in patients who subsequently die [30] [54]. This is the case of troponin I, which in some more seriously ill patients increases during hospitalization and immediately before death [53]. In contrast, certain markers are found higher in severe cases compared to mild cases but they usually do not exceed the reference values (Table 2).

The elevation of serum levels of inflammatory markers is commensurate with the severity of the disease and in some cases increases up to 12 times the reference value, possibly being related to the direct damage to the hepatic tissue or as a secondary effect of the pharmacological treatment used during the hospitalization of patients that evolve toward liver injury [50]. For this reason, it is recommended at least to determine the levels of ALT, bilirubin and albumin during the treatment of patients with hepatotoxic medication and those with preexisting hepatic conditions [72].

Also, the combination of eosinopenia and increased high-sensitivity C-reactive protein (hs-CRP) can serve to distinguish between patients suspected of presenting COVID-19 (supporting the diagnostic process) from patients with pneumonia or a respiratory infection similar to COVID-19 [73]. Moreover, elevated LDH is one of the most frequently altered biochemical parameters on admission [15] and the increase of certain inflammatory cytokines (IL-2, IL-6, TNFα) during the progression stage [74] can contribute information to the follow-up of the disease (Table 1). In prior cases of pneumonia caused by SARS-CoV infection (2003), it was found that the serum levels of pro-inflammatory cytokines (IFN-γ, IL-1, IL-6, IL-12, and TGF-dv) and chemokines (CCL2, CXCL9, CXCL10 and IL-8) in patients infected with SARS-CoV were higher than in healthy patients, while the level of the cytokine synthesis inhibitory factor (IL-10) in seriously ill patients was significantly lower than in healthy counterparts [75]. In a meta-analysis of COVID-19, several differences were observed between groups of more severe cases. The non-survivors in comparison with the survivors had significant increases in their leukocyte, total bilirubin, creatine kinase, serum ferritin and interleukin 6 (IL-6) values and more significant decreases in their lymphocyte and platelet counts [76].

Concerning pancreatic function, an elevation of amylase and lipase has been associated with severe cases, which is consistent with pancreatic injury evidenced by computed tomography in seriously ill patients [65].

The literature analysis reveals several limitations, in that the evidence is still limited to one population and could thus exclude relevant factors associated with the epidemiological characteristics, the virulence of different strains, mutations in the viral genome, sociocultural behavior, and socioeconomic and hygiene-sanitary conditions of other regions that could influence on the impact of the disease in different populations [77]. We hope that there will soon be more scientific evidence available to reflect these differences and sources of variability. Other limitations noted were that some of the studies analyzed [22] [25] [31] [29] did not include the reference ranges of their population [24], a follow-up of the biomarkers was not always carried out during the course of the hospitalization time, or there was no information available.

Interestingly, the use of other analytical technologies such as proteomic studies has enhanced the identification of potential biomarkers that express differentially depending on the severity of the clinical condition of COVID-19 patients, which include factors of the complement, coagulation factors, modulators of inflammation and pro-inflammatory factors. These findings, as a complement to the variations in classic laboratory parameters, could contribute to identify potential therapeutic targets against infectious agents [78].

Previous reports suggest that COVID-19 patients with different complications (inflammation, co-infection and thrombosis) can be categorized by analytical patterns, where personalized therapy could contribute to lower early mortality rates (OR 0.144; CI: 0.03-0.686; p = 0.015) in patients, thus supporting the idea that each situation requires a therapeutic focus adjusted to the type of altered pattern [79].

In conclusion, clinical laboratories play a pivotal role in the SARS-CoV-2 pandemic, not only from a diagnostic point of view but also in terms of the prognosis of COVID-19 patients, determining the degree of metabolic disorder of the patients and favoring the development of support tools for clinical decision making in order to adjust the therapy to the biological changes experienced by the subjects. Likewise, the laboratory work allows optimizing the hospital environment resources of the critical units of the health systems, resulting in the enhancement of the response time and efficiency of this response [17]. However, these approaches should be constantly reassessed based on new and reliable evidence published around the world, besides the incorporation of new technologies into the clinical laboratory work to obtain greater precision in the search for biochemical markers.

## Acknowledgements

Supported by FEQUIP2019-CS-05 and VIPUCT N°2016PF-PL-04 grant from the Vicerrectoría de Investigación y Postgrado, Universidad Católica de Temuco.

## Conflict of interest statement

All the authors declare that they have no conflict of interest in this work.

## List of abbreviations

ACE, angiotensin-converting enzyme; ACE2, angiotensin II-converting enzyme; ALT, alanine aminotransferase; ARDS, acute respiratory distress syndrome; ARI, acute renal insufficiency; ARL, acute renal injury; AST, aspartate aminotransferase; BUN, blood urea nitrogen; CK-MB, creatine kinase-MB; COVID-19, 2019 coronavirus disease; CRP, C-reactive protein; CT, computed tomography; DIC, disseminated intravascular coagulation; eGFR, estimated glomerular filtration rate; ESRD, end-stage renal disease; G-CSF, several other biomarkers such as granulocyte colony-stimulating factor; hs-CRP, high-sensitivity C-reactive protein; hsTn, high-sensitivity troponin; ICU, Intensive Care Unit; IFN, Interferon inducible protein 10; IL-, Interleukin; LDH, Lactate dehydrogenase enzyme; MAS, macrophage activation syndrome; MCP-1, monocyte chemoattractant protein 1; MOF, multiple organ failure; N8R, Neutrophil-to-CD8+ T cell ratio; NLR, Neutrophil-tolymphocyte ratio; NT-proBNP, N-terminal pro B type natriuretic peptide; OR, Odds ratio; RAAS, renin-angiotensin-aldosterone system; RBD, receptor-binding domain; SARS-CoV-2, coronavirus 2 of the severe acute respiratory syndrome; TBIL, total serum bilirubin; TNF, tumor necrosis factor; ULN, upper limit of normal.
